# Dietary assessment methods in epidemiological research: current state of the art and future prospects

**DOI:** 10.12688/f1000research.10703.1

**Published:** 2017-06-16

**Authors:** Androniki Naska, Areti Lagiou, Pagona Lagiou

**Affiliations:** 1 Department of Hygiene, Epidemiology and Medical Statistics School of Medicine, National and Kapodistrian University of Athens, 75 M. Asias Street, Goudi, GR-115 27, Athens, Greece; 2 Department of Public Health and Community Health,, School of Health Professions, Athens Technological Educational Institute (TEI Athens), Ag. Spyridonos, Aigaleo GR-122 43, Athens, Greece; 3 Department of Epidemiology, Harvard T.H. Chan School of Public Health, 677 Huntington Avenue, Boston, MA-02115, USA

**Keywords:** dietary questionnaire, nutritional epidemiological investigation, dietary intake assessment

## Abstract

Self-reported dietary intake is assessed by methods of real-time recording (food diaries and the duplicate portion method) and methods of recall (dietary histories, food frequency questionnaires, and 24-hour dietary recalls). Being less labor intensive, recall methods are more frequently employed in nutritional epidemiological investigations. However, sources of error, which include the participants’ inability to fully and accurately recall their intakes as well as limitations inherent in the food composition databases applied to convert the reported food consumption to energy and nutrient intakes, may limit the validity of the generated information. The use of dietary biomarkers is often recommended to overcome such errors and better capture intra-individual variability in intake; nevertheless, it has its own challenges. To address measurement error associated with dietary questionnaires, large epidemiological investigations often integrate sub-studies for the validation and calibration of the questionnaires and/or administer a combination of different assessment methods (e.g. administration of different questionnaires and assessment of biomarker levels). Recent advances in the omics field could enrich the list of reliable nutrition biomarkers, whereas new approaches employing web-based and smart phone applications could reduce respondent burden and, possibly, reporting bias. Novel technologies are increasingly integrated with traditional methods, but some sources of error still remain. In the analyses, food and nutrient intakes always need to be adjusted for total daily energy intake to account for errors related to reporting.

## Introduction

Adequate exposure assessment is a prerequisite in all epidemiologic investigations and presents particular challenges in studies of nutritional epidemiology. Diet represents an unusually complex exposure with strongly inter-correlated components. Early efforts to understand diet–disease associations focused on the role of specific nutrients, but later on it became evident that in several instances dietary exposures may act synergistically ^1^. Moreover, our eating habits may not only affect the way our genetic disposition is expressed but also probably participate in interplay with other lifestyle factors, such as physical activity and smoking ^2^. Trying to record what people eat is not an easy task, and, even when the best possible method or combination of methods are selected, some measurement error is introduced and needs to be accounted for in the analysis and interpretation of results ^3^. Adequate dietary intake assessment is important not only in the study of associations between diet and health-related outcomes but also for nutritional surveillance and the evaluation of the nutritional status of patients in clinical settings. In the following paragraphs, we will refer to the most commonly used methods of self-reported dietary intake and we will review biomarkers of diet. We will also discuss sources of errors in the assessment of dietary intake as well as approaches to address them.

## Methods of self-reported dietary intake

Methods of self-reported dietary intake can be grouped into two broad categories: methods of real-time recording and methods of recall. Real-time recording methods consist of food diaries (with or without weighing of foods consumed) and the duplicate portion method. When using food diaries, individuals are asked to record every food or beverage they consume in real time; in food diaries with weighing, individuals are also required to record the actual quantities consumed. In the duplicate portion method, pairs of daily portions are used: one is consumed by the individual, and the second is chemically analyzed for content. Because of their cost and complexity, the demanding duplicate portion method and the dietary diaries are less often used in large-scale epidemiologic investigations of diet–disease associations ^4^.

Methods of recall include dietary histories, food frequency questionnaires (FFQs), and single or multiple daily recalls (24-hour dietary recalls [24-HDRs]). In the vast majority of the nutritional epidemiology literature, two dietary assessment methods prevail: the FFQs, mainly in studies assessing diet and disease associations, and the 24-HDRs, mostly in nutrition surveillance studies, which monitor the populations usual intake and can help identify subpopulations in need of dietary guidance.

The FFQ relies on the principles of the diet history method. It records the frequency by which an individual consumes foods and beverages—listed either collectively (e.g. green leafy vegetables) or individually (e.g. lettuce)—over a long period of time, most frequently a year. The FFQ may or may not include questions on the usual quantity consumed (semi-quantitative, quantitative or non-quantitative FFQs, respectively); information on quantities is often collected with the use of photographs of various portions and household or standard units. The questionnaire can be administered by an interviewer or filled in directly by the participant, who is usually asked to indicate the frequency of consumption through pre-determined options. A short FFQ may underestimate the true variation in dietary intake, but a very long and detailed one can be time and resource consuming and the burden on the responder may jeopardize data quality. Researchers balance their FFQ choices between these extremes so as to cover foods of their particular interest adequately but also to include enough information that would allow them to estimate the individual’s total daily energy intake ^5^.

In a 24-HDR, participants are asked to recall and describe in detail and in an open-ended manner the foods and beverages consumed over one day, preferably the day before. Data collection can be either interviewer- or self-administered and it often follows the psychometric principles of structured, multi-faceted interviews that facilitate participants in their descriptions ^6^. The use of technology further allows the integration of databases assisting the recording of the type and quantity of foods consumed. These databases include, for instance, specific information on recipe ingredients, packaging material, products’ characteristics (e.g. low-fat or vitamin D fortified) or even brand names, as well as multiple portion size measurement aids. However, since one or a selection of individual days may not be representative of a person’s usual diet, studies employing this assessment method should be carefully designed to include multiple administrations and cover both seasonal and weekly variations in intake ^5^.

Sources of error are inherent to both FFQs and 24-HDRs. Both are prone to recall bias, particularly FFQs, since individuals are asked to report their intake retrospectively and usually refer to prolonged periods of time. Additionally, both can be hampered by the individual’s intentional misreporting of their consumption of certain foods, which can be affected by their personal characteristics (e.g. age, gender, overweight, or obesity) and could result in a differential misclassification with unpredicted consequences in the estimated associations ^7^. Other errors in the assessment of diet through FFQs and 24-HDRs can be introduced via the use of food composition databases to calculate energy, nutrient, and alcohol intake; natural variations or limited information regarding the composition of processed and packaged products as well as foods prepared out of home are the main culprits ^8^.

## Biomarkers of dietary intake

A biomarker is a biological specimen that serves as an indicator of intake or metabolism of dietary constituents or an indicator of nutritional status ^9, 10^.

A classification distinguishes biomarkers into recovery, concentration, replacement, and predictive biomarkers, although some biomarkers can fall into more than one of these categories ^11^.

- Recovery biomarkers are based on precise and quantitative knowledge of the physiological balance between intake and output and can provide a dose–response relationship with intake. They are sensitive and time-dependent and are not substantially affected by inter-individual differences in metabolism. They are often used as reference measures to validate self-reported intakes. Only a few recovery biomarkers are currently known, the best examples being doubly labeled water (DLW), which is used to measure total energy expenditure, and urinary nitrogen and potassium, which are used to estimate total daily protein and potassium intake, respectively.
- Concentration biomarkers are correlated with dietary intake, but they are affected by an individual’s metabolism or characteristics (e.g. age, gender, smoking habits, obesity, and daily physical activity). Therefore, they cannot be considered as surrogate measures of absolute intake and are less suitable than are recovery biomarkers for assessing the relative validity of dietary questionnaires, although they have been used in this context. Concentration biomarkers can be used in studies of association with disease risk. Examples of concentration biomarkers are fatty acids measured in adipose tissue or vitamins in blood, including carotenoids.
- Replacement biomarkers are similar to the concentration biomarkers but refer to compounds with limited information in food composition databases, or biomarkers of metabolic response to a dietary stimulus. Examples include aflatoxins and some phytoestrogens.
- Predictive biomarkers ** resemble the recovery ** biomarkers, as they are sensitive to intake in a dose–response manner, but their overall recovery is lower. The 24-hour urinary fructose and sucrose fall into this category ^12^.

Biomarkers can also be categorized into short-term (reflecting intake over hours/days and usually measured in urine, plasma, or serum), medium-term (reflecting intake over weeks/months and usually measured in red blood cells or adipose tissue), and long-term biomarkers (reflecting intake over months/years, and usually measured in hair, nails, or teeth) ^10^.

In addition to the “traditional” dietary biomarkers, the field of omics is opening up new perspectives for identifying novel biomarkers. Hence, in the context of genomics, ** polymorphisms in the lactase gene, for instance, can be used to reflect milk consumption in Mendelian randomization analyses (these analyses rely on the assumption that genotype distribution is unrelated to confounders and use variation in genes of known function to examine the effect of a modifiable exposure on disease). Similarly, in epigenomics, we can examine if the epigenetic regulation of specific genes can be affected by food intake; in transcriptomics, we can compare differences in the gene expression profile among individuals following different dietary patterns; and in lipidomics, proteomics, and metabolomics, we can compare differences or changes in the respective profiles cross-sectionally or following particular dietary interventions ^10^.

The use of dietary biomarkers is often recommended to overcome the errors of self-reported dietary intake and the bias introduced by the use of food composition tables ^11^. Nevertheless, using biomarkers to assess dietary intake also has its limitations ^13^. Several inter-individual factors can operate and generate variation in biomarker levels, which does not reflect solely differences in dietary intake. Hence, in addition to the individual’s gender and age, tobacco smoking, alcohol consumption, medication, and physical activity can also affect the measurement ^14^. Furthermore, interactions between dietary components, the type and handling of the biological samples (e.g. conditions related to blood drawing or urine collections and sample transport and storage), and the characteristics of the laboratory methods used (precision, detection limits, and inter-laboratory variations) may also contribute to the observed variations ^10, 13^. For these reasons, the use of a biomarker in nutritional epidemiology should be preceded by an assessment of its validity, reproducibility, ability to detect changes, and suitability for the population under study. According to recent reviews ^10, 14^, however, the majority of studies concentrate on the validity of biomarkers, whereas much less attention has been paid to the evaluation of their reproducibility and sensitivity in detecting changes in intakes and over time. In 2013, the US National Institute of Child Health and Human Development, supported by a consortium of NIH Offices and Divisions as well as the private sector, launched the Biomarkers of Nutrition for Development (BOND) program ^15^. This program aims to harmonize aspects related to dietary biomarkers and provide advice to researchers, clinicians, and policy makers on the process of making decisions about their best use in individual situations. In 2014, the Food Biomarkers Alliance (FoodBAll) project was launched in Europe with the support of the EU Joint Programming Initiative “A Healthy Diet for a Healthy Life”. The program aims to develop strategies for food intake biomarker discovery and validation, and to identify and validate biomarkers for a range of foods consumed across Europe ( http://foodmetabolome.org/).

## Dealing with errors in dietary intake assessment

Well-designed studies on diet–disease associations often provide inconsistent findings. Over the years, the discussion has evolved on whether this could also, at least partially, reflect limitations of the dietary assessment methods that generate measurement error of different magnitude. Kipnis and colleagues ^16^ described two potential components of the dietary measurement error. The first one reflects the correlation between error and true intake (“intake-related” bias), while the second is independent of true intake and represents errors related to the participant’s personal characteristics (“person-specific” bias). Measurement error can be systematic or random. Systematic errors reflect methodological weaknesses, appear at the group level, and generate differential misclassification. Random errors occur at the individual level, generate non-differential misclassification, and generally attenuate relative risk estimates and reduce statistical power to detect them ^3^. That said, even random measurement error can lead to biases towards or away from the null depending on multiple factors ^17, 18^.

### Energy adjustment

The US National Cancer Institute Observing Protein and Energy Nutrition (OPEN) study used intake biomarkers (DLW and urinary nitrogen) to evaluate the extent of misreporting and assess the components of the error introduced when a FFQ or a 24-HDR are collected on two occasions in a large sample of free-living individuals ^19^. According to their results, underreporting of energy was greater than that of protein, possibly indicating a preferential underreporting of fat, carbohydrate, and alcohol. The extent of underreporting was positively associated with intake and higher in the second administration of methods, reflecting probably the gradual loss of commitment after multiple administrations of time-demanding questionnaires. Underreporting, when present, affected all food groups, but the magnitude of underreporting varied between foods ^20^.

Though the impact of both the FFQ and the 24-HDR measurement error on total energy and absolute protein intakes was severe, it was substantially weakened after energy adjustment was applied ^16, 19^.

A more recent pooled analysis of data from five large validation studies conducted in the US (the Validation Studies Pooling Project) provided additional evidence on the nature and magnitude of reporting errors in FFQs and 24-HDRs by using assessments of DLW and three biomarkers (urinary nitrogen, potassium, and sodium) as surrogate measures of respective intakes. This pooled analysis confirmed that the impact of measurement error is weakened and estimates of relative risks are improved when energy adjustment is applied ^21, 22^.

With reference to evaluations of the observed energy intakes using the continuing US National Health and Nutrition Examination Survey (NHANES) data, it has been noted that, because of underreporting, the energy intake data could not be physiologically plausible for the majority of respondents ^23, 24^. It has also been acknowledged that alterations in measurement protocols and estimation procedures have led to improvements in the validity of more recent data collections and that these changes may have impaired the comparability of the findings over time, thus limiting our ability to estimate population trends in energy intake ^25^. Further assertions on the validity and usefulness of self-reported energy intake ^26^ and consequently the results of observational nutritional epidemiology studies have provoked discussion and replies. Commentaries tried to warn against a simplifying approach to address a genuinely complex issue and noted the results of high-quality studies that employed recovery biomarkers to assess intake and which consistently indicate that the estimates of self-reported intakes improve when values are adjusted for total energy intake ^27– 30^.

### Validation and calibration of dietary questionnaires

As already indicated, because of measurement error in self-reported intakes, an important association between diet and disease may be obscured. To address this issue, researchers involved in large epidemiological investigations integrate substudies for the validation and calibration of the dietary questionnaires. Since a gold-standard measurement in nutritional epidemiology is still lacking, a “validation study” aims to understand the structural equation of the measurement error model rather than assess the validity of an instrument measuring dietary intakes.

On the other hand, a “calibration study” aims to calculate correction factors (the attenuation factor) that will be applied to relative risk estimates derived with the administration of the dietary assessment method under calibration ^31^. A calibration study includes the simultaneous application of, presumably, a more accurate reference method (for instance, a biomarker of intake). The comparison of the reference measurements with those from the self-reported intakes provides the factors needed to adjust for attenuation through the regression calibration approach ^32– 34^. This approach, however, requires two critical assumptions about the independence of measurement errors. In particular, the reference method may contain measurement error itself, but this error has to be independent of true intake and of the error in the dietary questionnaire. Of note, in the OPEN study, the bias present in the 24-HDR measurements was correlated with the person-specific bias in the FFQ; correlation of these biases indicates that the 24-HDRs cannot necessarily be considered as an appropriate reference instrument for assessing the relative validity of a FFQ ^16, 19^.

### Combination of different dietary assessment methods

The administration of a combination of different assessment methods is becoming increasingly popular, as it can address several methodological limitations ^35, 36^. These methods can be objective, such as levels of dietary biomarkers, and/or subjective, such as self-reported intakes. For instance, a practice in nutrition surveillance surveys includes the administration of multiple 24-HDRs (preferably two per participant) together with a non-quantitative FFQ (often referred to as food propensity questionnaire) in order to estimate and remove the effects of within-person variation in dietary intake. The subsequently applied statistical modeling allows for the correlation between the probability of consuming a food (as reported in the non-quantitative FFQ) and the amount consumed on a particular day (as recorded through the two 24-HDRs) and further incorporates co-variate information relating to 24-HDRs ^37^.

### Repeated administration of methods assessing short-term intakes

Among dietary assessment methods, food diaries and 24-HDRs capture short-term dietary intakes. Between the two, 24-HDRs are more frequently used in large-scale nutritional epidemiology studies. Because of the day-to-day variability in intake, a single 24-HDR administered in a sufficiently large population sample can adequately provide data to estimate population mean intakes but fails to correctly depict the fraction of the population with usual intakes at the tails of the distribution. In addition, according to the results of the Validation Studies Pooling Project, multiple 24HDRs not only increased correlation with “true” intake compared with a single 24HDR but also substantially decreased attenuation of the relative risk estimates ^21, 22^.

Earlier efforts suggested the administration of multiple (usually two to seven) 24-HDRs and their averaging. This approach, however, bears other limitations given the high respondent burden and the consequent loss of data quality. Moreover, estimating the usual intake of episodically consumed foods based on a limited number of 24-HDRs per respondent presents additional challenges ^38^. To address these issues, methods based on the concurrent administration of different dietary questionnaires together with the collection of biological specimens to assess biomarkers of intake as well as the application of sophisticated statistical modeling are currently in use ^39^.

### Integration of new technology

In addition, novel technologies are integrated with traditional dietary assessment methods in an attempt to reduce the respondent’s burden and recall bias and to improve accuracy. These technological advances include computer software and web-based applications that aim to standardize the process of an interviewer-administered or a self-administered dietary report. As long as internet connection is available, free web-based tools can be accessed at any location, providing real-time data, reducing the impact of inconsistencies related to erroneous data entry, and allowing the automatic calculation of energy and nutrient intakes. The computer-based questionnaires often offer tutorials, are faster (since the skip patterns alleviate certain questions and detail questions show up only when something is consumed), probe into multiple details of the consumption in a harmonized manner, and provide digital images for food identification and portion-size estimation. In addition, mobile phone applications mitigate issues related to internet connection problems and make use of the phone camera and card to record consumption through sending digital images before and after eating. More advanced applications used in Japan and Australia combine imaging with voice recording. Study participants take photographs of foods and beverages before and after consumption and verbally describe them. They then upload the images and the voice files to the study website to be accessed by the researchers ^40^.

While the feasibility of multiple 24-HDRs combined with other dietary questionnaires has advanced with these new technologies, there are still important considerations related to the possibility of selection bias, since these methods cannot be applied to population subgroups who are not familiar with innovative technologies or modern devices. Furthermore, technical problems (e.g. internet connection of low quality) are still in need of improvement before these new methods become common practice. Although automated procedures may have benefits, they do not seem to overcome the limitations of self-reporting and the subjects’ inclination to intentionally misreport their intakes of particular food items. Hence, according to a recent report, even with the use of novel technologies, subjects still had difficulties in reporting their diet, they still underreported in repeated assessments, and, most importantly, they still altered their eating choices in response to being surveyed (social desirability bias) ^4^

## Conclusion

In nutritional epidemiology, several methods for the assessment of dietary intake are available, but they all have their own limitations. To overcome possible sources of error, current practice recommends the concurrent administration of different dietary questionnaires and the collection of biological specimens to estimate levels of dietary biomarkers. Recent advances in the omics field that could enrich the list of reliable nutrition biomarkers as well as novel technologies applied to reduce respondent burden and reporting bias will open up new prospects in the field. In the analyses, food and nutrient intakes always need to be adjusted for total daily energy intake to account for errors related to reporting. Moreover, dietary pattern analyses are increasingly applied; they have shown consistent findings across cohorts and mitigate the issues related to food composition tables. Notwithstanding the limitations in exposure assessment, nutritional epidemiology has generated evidence which has contributed to the formulation of public health policies and related measures ^30, 41^.

## Abbreviations

24-HDR, 24-hour dietary recall; DLW, doubly labeled water; FFQ, food frequency questionnaire; NHANES, National Health and Nutrition Examination Survey; NIH, National Institute of Health; OPEN, Observing Protein and Energy Nutrition.
